# Osteomyelitis Caused by *Moesziomyces aphidis* in an Immunocompetent Adult: A Case Report and Literature Review

**DOI:** 10.3390/tropicalmed10040107

**Published:** 2025-04-14

**Authors:** Yi-Ying Chen, Ting-Kuang Yeh, Chi-Jung Wu, Po-Yu Liu

**Affiliations:** 1 Department of Internal Medicine, Taichung Veterans General Hospital, Taichung 407219, Taiwan; 2 Division of Infectious Diseases, Department of Internal Medicine, Taichung Veterans General Hospital, Taichung 407219, Taiwan; 3 Genomic Center for Infectious Diseases, Taichung Veterans General Hospital, Taichung 407219, Taiwan; 4 National Institute of Infectious Diseases and Vaccinology, National Health Research Institutes, Tainan 704, Taiwan; 5 Doctoral Program in Translational Medicine, National Chung Hsing University, Taichung 402202, Taiwan; 6 Rong Hsing Translational Medicine Research Center, National Chung Hsing University, Taichung 402202, Taiwan; 7 Department of Post-Baccalaureate Medicine, College of Medicine, National Chung Hsing University, Taichung 402202, Taiwan

**Keywords:** *Moesziomyces*, ITS sequencing, osteomyelitis, amphotericin B, voriconazole

## Abstract

Osteomyelitis caused by *Moesziomyces aphidis* is exceedingly rare and, to our knowledge, has not been previously reported in immunocompetent individuals. This case represents the first documented instance. A 19-year-old woman developed osteomyelitis following an open right leg fracture sustained in a traffic accident. Initial cultures yielded an unidentified yeast, later identified as *M. aphidis* through internal transcribed spacer sequencing. The patient demonstrated clinical improvement with combination therapy of amphotericin B and following oral voriconazole. This case underscores the emerging pathogenic potential of *Moesziomyces* spp., particularly in the setting of trauma and open wounds, and highlights the importance of including this genus in the differential diagnosis of osteomyelitis. A literature review indicates that *Moesziomyces* infections are predominantly observed in immunocompromised patients, especially in Asia. However, our case underscores the need for greater awareness of this emerging pathogen in immunocompetent hosts as well.

## 1. Introduction

The genus *Moesziomyces (Ustilaginales*, *Ustilaginaceae)* was first described by Vánky in 1986 for smut fungi, and was characterized by ruptured sterile cells in the sori around the spores [1]. The species is mainly isolated from plant surfaces and provides a natural source of protection against powdery mildew. Wang et al. first reclassified four species previously identified solely by their anamorphs (*Pseudozyma antarctica*, *P. aphidis*, *P. parantarctica,* and *P. rugulosa*) into *Moesziomyces* [2]. Subsequently, eight species, including *Moesziomyces antarcticus*, *M. bullatus*, *M. globuligerus*, *M. kimberleyensis*, *M. parantarcticus*, *M. eriocauli*, *M. penicillariae* and *M. verrucosus*, were later classified into *Moesziomyces* spp. based on phylogenetic analyses [3,4,5]. Current reports of human infection by *Moesziomyces* spp. are relatively rare and often occur in immunocompromised patients or individuals on immunosuppressive therapy. Data on the clinical characteristics and pathogenicity of humans remain insufficient. Here, we present the first report of osteomyelitis associated with *Moesziomyces* spp. in an immunocompetent adult by internal transcribed spacer (ITS) sequencing and a literature review.

## 2. Case Presentation

A 19-year-old woman without systemic disease was admitted with pain in the right leg after a traffic accident. The patient, a college student, was otherwise healthy aside from obesity (BMI 31.1). The event took place when she was riding a scooter and fell into a ditch, falling onto the lateral aspect of her right ankle, resulting in an open fracture of the right leg with a large laceration wound about 15 cm. The avulsion wound was contaminated with the surrounding soil and mild cyanosis was observed. A knee computed tomography scan was performed with the finding of a fracture of the right proximal tibia shaft. Therefore, the patient underwent external fixation of the right proximal tibia shaft fracture, debridement, and partial closure of the wound with the placement of a drainage tube on the same day.

Following admission after surgery, her symptoms progressed to fevers of up to 38.1 °C. Osteomyelitis was diagnosed with intraoperative culture yielding *Serratia marcescens* and unidentified yeast species. Therefore, beginning on postoperative day 7, we initiated a regimen of ceftriaxone (2 g every 12 h) and liposomal amphotericin B (1.25 mg/kg/day). Simultaneously, wound drainage from the right tibia was collected and submitted for bacterial and fungal cultures. Bacterial cultures were negative for bacterial growth, but the fungal culture initially yielded an unidentified yeast. Subsequent identification revealed the isolate as *M. aphidis* (synonym: *M. bullatus*) based on a 100% (658/658) sequence identity of the internal transcribed spacer (ITS) region (GenBank ID: LC816133) compared with the *M. aphidis* strain CBS.517.83 (GenBank ID: NR_145336). The antifungal susceptibility testing results of the isolate are shown in Table 1.

Antifungal therapy with amphotericin B was maintained for 12 days. We changed amphotericin B to oral voriconazole 200 mg Q12H as a maintenance therapy for *Moesziomyces* infection. The patient was discharged in good condition and completed a total of six weeks of oral voriconazole therapy. The report was conducted according to the guidelines of the Declaration of Helsinki and approved by the Institutional Review Board of Taichung Veterans General Hospital (CE20004B).

## 3. Discussion

This report describes an immunocompetent patient with *Moesziomyces* osteomyelitis. Due to the limitation of clinical diagnostic tools, it was challenging to diagnose this fungal infection without performing PCR or genome sequencing. Given the limited number of reported human infections with *Moesziomyces* species, further studies are needed to better understand its clinical relevance and pathogenicity. Nevertheless, the advent of PCR assays and DNA sequencing technology has improved its identification and allowed for more accurate diagnosis by combination with microscopic examination, fungal culture, and clinical symptoms. While genomic sequencing is increasingly available in high-resource settings, its implementation in low-resource environments remains limited. Tailored, context-specific approaches will be necessary to integrate molecular diagnostics into clinical practice in these settings [6].

To gain a deeper understanding of *Moesziomyces* species infections in humans, we conducted a literature review of clinical reports published before 31 January 2025. The search was performed using PubMed, Google Scholar, and the English-language version of the Web of Science database. Keywords included “*Moesziomyces*”, “*Pseudozyma*”, and “fungal infection”. A total of 16 cases were identified and summarized, based on the availability of clinical information and/or antifungal susceptibility data. The etiological agents were identified by morphology and molecular methods, and the strain information and clinical details were retrieved, as shown in Table 2 [7,8,9,10,11,12,13,14,15,16,17,18,19,20,21,22]. About half of the cases were reported from Asian countries (7/16, 43.7%), particularly from Northeast Asia. A total of four species were reported, among which *M. aphidis* is most frequent (10/16, 62.5%). It is well known that immunocompromised individuals are more susceptible to invasive fungal infections. In our review, most patients (14/16, 87.5%) had either systemic immunodeficiency—such as prematurity, low birth weight, Crohn’s disease, short gut syndrome, malignancy, or chemotherapy—or a disruption of local barriers, including impaired skin integrity due to dermatoses or surgical wounds, and gastrointestinal breaches such as those caused by peritoneal dialysis. Only two cases eventually died. About 62.5% (10/16) of these strains were isolated from blood samples, and the others were isolated form peritoneal dialysis fluid, hand, vitreous aspirate, leg, pleural fluid, and abscess of brain, respectively. In this report, *Moesziomyces aphidis* was isolated from the wound of an immunocompetent patient, suggesting a novel clinical presentation. Fungal osteomyelitis is rare and often indolent, typically resulting from direct inoculation through trauma, surgery, or contaminated wounds. As fungal pathogens are seldom considered early in post-traumatic infections, clinicians should keep a broader differential in cases unresponsive to standard antibacterial therapy [23].

The antifungal therapy and treatment duration for *Moesziomyces* species is still uncertain. Hence, we also summarized the available antifungal drug resistances data and treatment duration in Table 3 [7,8,9,10,11,12,13,14,15,16,17,18,19,20,21,22]. Most strains had low MICs for itraconazole, voriconazole, amphotericin B, and posaconazole, while high MICs for fluconazole (0.5 → >256 mg/L), flucytosine (>32 mg/L), caspofungin (4 → >32 mg/L), anidulafungin (4 → >8 mg/L), and micafungin (>8 mg/L). The treatment duration varies from 2 weeks to 1 year. The high MIC values for fluconazole and itraconazole observed in this case draw attention to the potential variability in antifungal susceptibility among rare yeast pathogens. Although definitive conclusions cannot be made based on a single isolate, such findings reinforce the importance of maintaining clinical vigilance when encountering unidentified fungal organisms. In this context, phenotypic susceptibility remains a valuable tool in selecting appropriate antifungal regimens.

One systemic review of fungal osteomyelitis reported that the median duration of treatment was 4 months and that the most common strategies were amphotericin B and azoles [24]. In this case, the fungus had high MICs for fluconazole and itraconazole, a low MIC for amphotericin B (0.5 μg/mL), and an intermediate MIC for voriconazole (2 μg/mL). The patient received amphotericin B for 12 days followed by oral voriconazole and clinically improved without complications.

## 4. Conclusions

In this paper, we report the first bone tissue infection caused by *Moesziomyces* spp. and provide a literature review of human disease associated with *Moesziomyces* fungi. By organizing strain information and clinical presentations, we shed light on its geographical distribution, risk factors, and susceptibility to routine antifungal agents. It is crucial that physicians employ optimal diagnostics to identify possible pathogens and choose the most appropriate antifungal regimens. Notably, the patient in this case responded well to targeted antifungal therapy with amphotericin B and voriconazole, guided by susceptibility testing. This case further underscores the importance of maintaining diagnostic vigilance in post-traumatic infections, where uncommon fungal pathogens such as *Moesziomyces* may be overlooked.

## Figures and Tables

**Table 1:** Antifungal susceptibility of antifungal agents to *Moesziomyces aphidis* according to the CLSI M38A3 guidelines.

Antifungal Agent	MIC (μg/mL)	MEC (μg/mL)
Amphotericin B	0.5	
Itraconazole	>16 ^#^	
Voriconazole	2	
Posaconazole	1	
Isavuconazole	2	
Ketoconazole	0.5	
Anidulafungin	>8	<0.004
Terbinafine	8	
Fluconazole	>256	

Abbreviations: MEC, minimum effective concentration; MIC, minimum inhibitory concentration. ^#^ A 50% reduction in fungal growth was achieved in wells containing itraconazole at concentrations ranging from 0.03 to 16 μg/mL.

**Table 2:** Information about the cases and strains of *Moesziomyces* or *Pseudozyma* species in human.

Study	Country	Reported Strain	Age	Gender	Risk	Diagnosis	Source	Final Method of Identification	Outcome
Sulik-Tyszka et al., 2025 [7]	Poland	*Moesziomyces aphidis*	88	Male	Pancreatic cancer	Bacteremia	Blood	MALDI-TOF MS	Resolved
Nishioka et al., 2022 [8]	Japan	*Moesziomyces antarcticus*	70	Male	ESRD	Peritonitis	PD fluid	ITS sequencing	Resolved
Mpakosi et al., 2022 [9]	Greece	*Moesziomyces aphidis*	0	Female	VLBW preterm	Sepsis	Blood	ITS sequencing	Resolved
Hu et al., 2021 [10]	China	*Dirkmeia churashimaensis* (*ustilaginales* spp.)	80	Female	N/A	Subcutaneous mycoses	Skin	ITS sequencing	Resolved
Liu et al., 2019 [11]	China	*Moesziomyces antarcticus*	93	Male	CKD, AD, cerebral infarction	Bacteremia	Blood	ITS sequencing	Resolved
Voona et al., 2019 [12]	New Zealand	*Pseudozyma aphidis*	46	Male	Cataract extraction surgery	Endophthalmitis	Vitreous aspirate	MALDI-TOF MS	Resolved
Joo et al., 2015 [13]	Korea	*Pseudozyma aphidis*	51	Male	AML	Pneumonia, FN	Blood	ITS sequencing	Resolved
Herb et al., 2015 [14]	France	*Pseudozyma aphidis*	68	Female	Metastatic adenocarcinoma of the ampulla vater	Sepsis	Blood	ITS sequencing	N/A
Okolo et al., 2015 [15]	Nigeria	*Moesziomyces bullatus*	0	Female	LBW preterm	Neonatal sepsis	Blood	ITS sequencing	Expired
Orecchini et al., 2015 [16]	Argentina	*Pseudozyma aphidis*	6	Female	Osteosarcoma	Febrile neutropenia	Blood	ITS sequencing	Resolved
Siddiqui et al., 2014 [17]	USA	*Pseudozyma* spp.	52	Female	Crohn’s disease	Fever	Blood	ITS sequencing	Resolved
Prakash et al., 2014 [18]	India	*Pseudozyma aphidis*	0	Male	LBW full- term	Neonatal jaundice	Blood	Sequencing of LSU	Resolved
Parahym et al., 2013 [19]	Brazil	*Pseudozyma aphidis*	17	Male	Burkitt lymphoma, chemotherapy	Pneumonia, FN	Pleural fluid	ITS sequencing	Clinical improvement
Chen et al., 2011 [20]	China	*Pseudozyma aphidis*	51	Male	N/A	Leg mycetoma	Pus	ITS sequencing	Resolved
Hwang et al., 2009 [21]	Korea	*Pseudozyma* spp.	78	Male	Astrocytoma	Brain abscess	Abscess	ITS sequencing	Expired
Lin et al., 2007 [22]	USA	*Pseudozyma aphidis*	7	Female	Short gut syndrome	Fever	Blood	ITS sequencing	Resolved
This study	Taiwan	*Moesziomyces* spp.	19	Female	N/A	Osteomyelitis	Wound	ITS sequencing	Clinically improved

ESRD, end-stage renal disease; PD, peritoneal dialysis; ITS, internal transcribed spacer; LSU, large subunit; FN, febrile neutropenia; VLBW, very low birth weight; CKD, chronic kidney disease; AD, Alzheimer’s disease; MALDI-TOF, matrix-assisted laser desorption/ionization time of flight; AML, acute myeloid leukemia; LBW, low birth weight; N/A, not available.

**Table 3:** MIC and treatment durations of *Moesziomyces* or *Pseudozyma* spp. isolated from human infection.

Study	Strain	MICs (mg/L)									Treatment Regimen, Duration
		FLC	ITC	VRC	AMB	CAS	5FC	ANI	POS	Others	
Sulik-Tyszka et al., 2025 [7]	*Moesziomyces aphidis*	>256	N/A	0.125	0.125	>8	N/A	>8	N/A	MCFG >8	Oral VRC, 2 weeks
Nishioka et al., 2022 [8]	*Moesziomyces antarcticus*	>64	0.25	0.5	1	16	>64	N/A	N/A	MCZ 8; MCFG >16	CAS→VRC, 8 weeks
Mpakosi et al., 2022 [9]	*Moesziomyces aphidis*	0.5	0.5	0.25	0.25	>8	>64	>8	2		LAMB, 3 weeks
Hu et al., 2021 [10]	*Dirkmeia churashimaensis* (*Ustilaginales* spp.)	64	4	2	0.5	N/A	64	N/A	2	KET 0.5	Oral ITC, 6 months
Liu et al., 2019 [11]	*Moesziomyces antarcticus*	128	4	8	<0.5	N/A	>64	N/A	N/A		CAS→AMB, 3 weeks
Voona et al., 2019 [12]	*Pseudozyma aphidis*	4	N/A	0.06	1	N/A	N/A	N/A	N/A		oral and intravitreal VRC, N/A
Joo et al., 2015 [13]	*Pseudozyma aphidis*	N/A	N/A	N/A	N/A	N/A	N/A	N/A	N/A		LAMB→VRC, 4 months
Herb et al., 2015 [14]	*Pseudozyma aphidis*	16	0.19	0.03	0.19	>32	>32	N/A	0.094		LAMB, 2 weeks
Okolo et al., 2015 [15]	*Moesziomyces bullatus*	128	0.12	0.03	1	8	64	8	0.03	MCFG 8	FLC, N/A
Orecchini et al., 2015 [16]	*Pseudozyma aphidis*	2	0.03	0.03	0.13	N/A	128	N/A	0.015		LAMB, 2 weeks
Siddiqui et al., 2014 [17]	*Pseudozyma* spp.	N/A	N/A	N/A	N/A	N/A	N/A	N/A	N/A		N/A, N/A
Prakash et al., 2013 [18]	*Pseudozyma aphidis*	8	0.03	0.06	0.03	8	>64	8	N/A	ISA 0.25; MCFG >8	AMB→VRC, 2 weeks
Parahym et al., 2013 [19]	*Pseudozyma aphidis*	4	0.25	0.03	0.25	4	N/A	4	N/A		LAMB→VRC, 25 days
Chen et al., 2011 [20]	*Pseudozyma aphidis*	N/A	N/A	N/A	N/A	N/A	N/A	N/A	N/A		ITC, 1 year
Hwang et al., 2009 [21]	*Pseudozyma* spp.	N/A	N/A	N/A	N/A	N/A	N/A	N/A	N/A		N/A, N/A
Lin et al., 2007 [22]	*Pseudozyma aphidis*	4	0.125	N/A	0.25	N/A	N/A	N/A	N/A		FLC→ITC, 2 weeks
This study	*Moesziomyces* spp.	>256	>16	2	0.5	N/A	N/A	>8	1	VRC 2, ISA 2, KET 0.5, TRB 8	AMB→oral VRC, 6 months

FLC, fluconazole; ITC, itraconazole; VRC, voriconazole; AMB, amphotericin B; CAS, caspofungin; 5FC, flucytosine; LAMB, liposomal amphotericin B; ANI, anidulafungin; POS, posaconazole; MCZ, miconazole; MCFG, micafungin; KET, ketoconazole; ISA, isavuconazole; TRB, terbinafine; N/A, not available.

## Data Availability

The genomes have been deposited in GenBank with accession numbers LC816133. Further inquiries can be directed to the corresponding author.

## References

[1] Vánky K. (1986). The Genus Moesziomyces (Ustilaginales). Nord. J. Bot..

[2] Wang Q.-M., Begerow D., Groenewald M., Liu X.-Z., Theelen B., Bai F.-Y., Boekhout T. (2015). Multigene Phylogeny and Taxonomic Revision of Yeasts and Related Fungi in the Ustilaginomycotina. Stud. Mycol..

[3] Kruse J., Doehlemann G., Kemen E., Thines M. (2017). Asexual and Sexual Morphs of Moesziomyces Revisited. IMA Fungus.

[4] Tanaka E., Koitabashi M., Kitamoto H. (2019). A Teleomorph of the Ustilaginalean Yeast *Moesziomyces antarcticus* on Barnyardgrass in Japan Provides Bioresources That Degrade Biodegradable Plastics. Antonie Van Leeuwenhoek.

[5] Li Y.M., Shivas R.G., Li B.J., Cai L. (2019). Diversity of *Moesziomyces* (Ustilaginales, Ustilaginomycotina) on *Echinochloa* and *Leersia* (Poaceae). Mycokeys.

[6] Pronyk P.M., de Alwis R., Rockett R., Basile K., Boucher Y.F., Pang V., Sessions O., Getchell M., Golubchik T., Lam C. (2023). Advancing Pathogen Genomics in Resource-Limited Settings. Cell Genom..

[7] Sulik-Tyszka B., Małyszko J., Pęczuła A., Jarzynka S. (2025). *Moesziomyces aphidis* Bloodstream Infection in Oncologic Patient: First Report in Poland. J. Fungi.

[8] Nishioka S., Yamanaka M., Kunisho Y., Aoki Y., Higasitani M., Yokoyama T., Oyama T., Ohkusu M., Kamei K., Sofue T. (2022). A Case of *Moesziomyces antarcticus* Peritonitis in a Patient Undergoing Peritoneal Dialysis. Ren. Replace. Ther..

[9] Mpakosi A., Siopi M., Demetriou M., Falaina V., Theodoraki M., Meletiadis J. (2022). Fungemia Due to *Moesziomyces aphidis* (*Pseudozyma aphidis*) in a Premature Neonate. Challenges in Species Identification and Antifungal Susceptibility Testing of Rare Yeasts. J. Mycol. Med..

[10] Hu F., Wang C., Wang P., Zhang L., Jiang Q., Al-Hatmi A.M.S., Blechert O., Zhan P. (2021). First Case of Subcutaneous Mycoses Caused by *Dirkmeia churashimaensis* and a Literature Review of Human *Ustilaginales* Infections. Front. Cell. Infect. Microbiol..

[11] Liu Y., Zou Z., Hu Z., Wang W., Xiong J. (2019). Morphology and Molecular Analysis of *Moesziomyces antarcticus* Isolated from the Blood Samples of a Chinese Patient. Front. Microbiol..

[12] Voon S.M., Upton A., Gupta D. (2019). *Pseudozyma aphidis* Endophthalmitis Post-Cataract Operation: Case Discussion and Management. Am. J. Ophthalmol. Case Rep..

[13] Joo H., Choi Y.-G., Cho S.-Y., Choi J.-K., Lee D.-G., Kim H.-J., Jo I., Park Y.-J., Lee K.-Y. (2016). *Pseudozyma aphidis* Fungaemia with Invasive Fungal Pneumonia in a Patient with Acute Myeloid Leukaemia: Case Report and Literature Review. Mycoses.

[14] Herb A., Sabou M., Delhorme J.B. (2015). *Pseudozyma aphidis* Fungemia after Abdominal Surgery: First Adult Case. Med. Mycol. Case Rep..

[15] Okolo O.M., Van Diepeningen A.D., Toma B. (2015). First Report of Neonatal Sepsis Due to *Moesziomyces bullatus* in a Preterm Low-Birth-Weight Infant. JMM Case Rep..

[16] Orecchini L.A., Olmos E., Taverna C.G., Murisengo O.A., Szuzs W., Vivot W., Córdoba S., Bosco-Borgeat M.E., Montanaro P.C. (2015). First Case of Fungemia Due to *Pseudozyma aphidis* in a Pediatric Patient with Osteosarcoma in Latin America. J. Clin. Microbiol..

[17] Siddiqui W., Ahmed Y., Albrecht H., Weissman S. (2014). Pseudozyma spp Catheter-Associated Blood Stream Infection, an Emerging Pathogen and Brief Literature Review. BMJ Case Rep..

[18] Prakash A., Wankhede S., Singh P.K. (2014). First Neonatal Case of Fungaemia Due to *Pseudozyma aphidis* and a Global Literature Review. Mycoses.

[19] Parahym A.M., Da Silva C.M., Ide D. (2013). Pulmonary Infection Due to *Pseudozyma aphidis* in a Patient with Burkitt Lymphoma: First Case Report. Diagn. Microbiol. Infect. Dis..

[20] Chen B., Zhu L.-Y., Xuan X., Wu L.-J., Zhou T.-L., Zhang X.-Q., Li B.-X. (2011). Isolation of Both *Pseudozyma aphidis* and *Nocardia otitidiscaviarum* from a Mycetoma on the Leg. Int. J. Dermatol..

[21] Hwang S., Kim J., Yoon S. (2010). First Report of Brain Abscess Associated with *Pseudozyma* Species in a Patient with Astrocytoma. Korean J. Lab. Med..

[22] Lin S.-S., Pranikoff T., Smith S.F., Brandt M.E., Gilbert K., Palavecino E.L., Shetty A.K. (2008). Central Venous Catheter Infection Associated with *Pseudozyma aphidis* in a Child with Short Gut Syndrome. J. Med. Microbiol..

[23] Khan S., Kumar A., Bhaskaran V., Chandran S., Dinesh K. (2019). Chronic Fungal Osteomyelitis of the Tibia Due to *Acremonium curvulum*: A Rare Case. Pan Afr. Med. J..

[24] Asperges E., Albi G., Truffelli F. (2023). Fungal Osteomyelitis: A Systematic Review of Reported Cases. Microorganisms.

